# Anthocyanins from *Lycium ruthenicum* Murray Inhibit HepG2 Cells Growth, Metastasis and Promote Apoptosis and G2/M Phase Cycle Arrest by Activating the AMPK/mTOR Autophagy Pathway

**DOI:** 10.1155/2022/9609596

**Published:** 2022-12-30

**Authors:** Hongli Fan, Yonggan Ji, Yang Wang, Danni Liu, Tingting Wei, Xue Cao, Mengmeng Yang, Changcai Bai, Zhisheng Wang

**Affiliations:** ^1^School of Pharmacy, Ningxia Medical University, Yinchuan 750004, Ningxia Hui Autonomous Region, China; ^2^The Third Clinical College, Ningxia Medical University, Yinchuan 750004, Ningxia Hui Autonomous Region, China; ^3^Cancer Hospital, Ningxia Medical University General Hospital, Yinchuan 750004, Ningxia Hui Autonomous Region, China; ^4^Laboratory Animal Center, Ningxia Medical University, Yinchuan 750004, Ningxia Hui Autonomous Region, China

## Abstract

Among the most common malignancies in humans, liver cancer ranks third in terms of mortality in the world. Seeking new anticancer drugs or adjuvant chemotherapy drugs from natural products has attracted the attention of many researchers. *Lycium ruthenicum* Murray (LR), a health food and traditional Chinese medicine, exerts extensive pharmacological properties, of which anthocyanins are one of the key active components. In this research, we explored the antitumor activity and autophagy regulation mechanism of anthocyanins from *Lycium ruthenicum* Murray (ALR) in HepG2 cells. Our results found that ALR profoundly reduced the cell viability, clone formation, migration, and invasion and promoted apoptosis and G2/M phase arrest of HepG2 cells in a dose-dependent pattern. Further studies confirmed that ALR treatment significantly increased the number of autophagic vacuoles and autophagosomes, upregulated the expression of Beclin-1, p62, LC3-II/LC3-I, and p-AMPK, and concomitantly downregulated the expression of p-mTOR. When autophagy was inhibited by 3-methyladenine (3-MA), ALR-induced proliferation inhibition, invasion, and migration capabilities, as well as apoptosis rate and G2/M phase arrest, were all reversed, and the activities of key proteins in the AMPK/mTOR pathway were all constrained. In summary, the results presented here indicate that ALR may be effective as a natural antitumor agent by activating AMPK and inhibiting the mTOR autophagy pathway in HepG2 cells.

## 1. Introduction

The majority of liver cancers in the digestive system are hepatocellular carcinoma (HCC), which accounts for 75–85% of cases [1]. By 2030, liver cancer will cause approximately 1,000,000 deaths, according to the World Health Organization [2]. In clinical practice, surgery, chemotherapy, and radiotherapy are the conventional treatment approaches for HCC [3]. However, these treatments have limited efficacy for advanced HCC and have serious side effects due to their weak selectivity for cancer cells [4]. Hence, it is urgently necessary to develop efficient and safe new antitumor drugs.

Natural products are an important source for discovering anticancer or adjuvant chemotherapy agents. At present, many natural or mimicked natural products have been approved for clinical use as anticancer drugs, such as irinotecan, vincristine, etoposide, and paclitaxel [5]. LR is vividly known as “the king of anthocyanins” because of its high content of anthocyanins [6]. Its fruit has become an indispensable ingredient in people's daily diets [7]. Numerous researchers have noted that anthocyanins play a significant role in cancer prevention and management [8]. For example, anthocyanins derived from black chokeberries exhibited anticancer activity by preventing the growth, adhesion, and metastasis of SK-Hep1 cells [9]. In addition, anthocyanin supplements as a dietary strategy may possess potential anticancer activity by suppressing the viability of cancer cells, but epidemiological studies in humans still need further confirmation [10]. Mechanistically, the anticancer effects of anthocyanins are mainly involved in apoptosis and autophagy [11, 12]. Unlike apoptosis, which ultimately results in cell death, autophagy determines cell fate in a bidirectional manner, relying on the strength and duration of irritants [13]. Moderate autophagy can maintain cell homeostasis to promote cell survival, while excessive autophagy impairs essential cellular components and processes to activate apoptosis or necrosis, ultimately leading to cell death [14]. A study found that mulberry anthocyanins may be used as adjuvant therapy in patients with thyroid cancer by activating autophagic cell death [12], while another study confirmed that anthocyanins in black soybean prevented apoptosis by inducing autophagy in U2OS cells [15]. These studies reveal that anthocyanin-induced autophagy plays a completely different role in distinct tumors. A growing body of literature has demonstrated that the AMP-activated protein kinase (AMPK)/mammalian target of rapamycin (mTOR) signaling pathway plays an important role in the regulation of autophagy [16]. AMPK, an energy sensor, is a positive regulator of autophagy, and its activation can lead to decreased phosphorylation of mTOR, thereby promoting cellular autophagy [17]. Nevertheless, the exact role of ALR in HCC and whether it has a selective toxic or protective effect on HepG2 cells in terms of autophagy remain unclear. Therefore, it is crucial to clarify the role and regulatory mechanism of autophagy in ALR anti-HCC.

In this research, we first evaluated the antitumor properties of ALR in HepG2 cells and then further explored its possible antitumor mechanism. Our study is the first to demonstrate that ALR plays an anti-HCC role by inhibiting HepG2 cell proliferation and metastasis and promoting apoptosis and G2/M phase cycle arrest by activating the AMPK/mTOR autophagy pathway. According to our findings, ALR can be used as a potential candidate or adjuvant agent for treating HCC.

## 2. Materials and Methods

### 2.1. Reagents

ALR (anthocyanins 51.6%, ash 3.31%, moisture 4.58%, heavy metal <10 ppm, pesticide residues <1 ppm) was obtained from the Realin Biotechnology company (Shaanxi, Xi'an, China). 3-methyladenine (3-MA) was purchased from APExBIO Company (Houston, USA). DMEM medium and fetal bovine serum (FBS) were obtained from Gibco Company (SA, USA). LC3 (Q9GZQ8) was obtained from Abmart Company (Shanghai, China). p62 (ab56416), Beclin-1 (ab210498), AMPK (ab32047), p-AMPK (ab133448), mTOR (ab32028), p-mTOR (ab109268), and horseradish peroxidase (HRP)-labeled rabbit/mouse IgG H&L were obtained from Abcam Company (Cambridge, USA). *β*-Actin was obtained from ZSGB-BIO Company (Beijing, China).

### 2.2. Cell Culture and Sample Treatment

Human hepatocellular carcinoma cell lines (HepG2) and human normal hepatocyte cell lines (LO2) were bought from the Shanghai Institute of Cell Biology, China. Cells were grown in DMEM containing 10% FBS and 1% penicillin/streptomycin (P/S, Solarbio, Beijing, China) at 37°C and 5% CO_2_ humidified atmosphere (Thermo, Waltham, USA). The 3-MA was dissolved in serum-free DMEM medium to prepare 20 mM mother liquor, filtered, aliquoted, and stored at −20°C for subsequent assays. The ALR was refrigerated and packed in tin foil, dissolved in serum-free DMEM medium, and used immediately after aseptic filtration with a 0.22 *μ*m syringe filter.

### 2.3. Cell Viability Assay

HepG2 and LO2 cells were planted on a 96-well plate overnight and then stimulated with varying doses of ALR with or without 3-MA (2.5 mM) for another 24 or 48 h. After that, the CCK-8 reagent (Beyotime, Shanghai, China) was appended to each well (10 *μ*L/well), and then incubated for additional 30 min at 37°C, followed by measuring the optical density (OD) value at 450 nm using a microplate reader (Multiskan GO, Thermo, USA), with 5 duplicates in each group. Cell viability (%) = (OD_treatment_ − OD_blank_)/(OD_control_ − OD_blank_) × 100%.

### 2.4. Colony Formation Assay

HepG2 cells were planted on a 6-well plate overnight and then stimulated with varying doses of ALR for another 24 h. After that, the cells were grown in DMEM medium containing 0.5% FBS and 1% P/S for approximately 16 days until colonies were evident. Then, the colonies were fixed and colored using 4% paraformaldehyde and 0.1% crystal violet, respectively, and finally, pictures were captured under natural light.

### 2.5. Cell Scratch Assay

HepG2 cells were planted on a 6-well plate until the density reached 95%. Then, the cells were scratched using 200 *μ*L sterile tips and cultured for 24 h with different doses of ALR with or without 3-MA (2.5 mM). The wound width of each group was observed and captured under a microscope. By comparing the average area of migration to the starting scratch area, the migration ratio was calculated.

### 2.6. Cell Migration and Invasion Assays

After treatment with varying doses of ALR with or without 3-MA (2.5 mM) for 24 h, HepG2 cells were resuspended in DMEM medium without FBS and planted into a 24-well transwell (upper chamber, pore size 8 *μ*m, Corning) at a density of 2 × 10^4^ cells/well (precoated with 0.5 mg/mL Matrigel (Corning, NY, USA) for invasion assay, not for migration assay). The lower chamber was filled with the DMEM complete medium. After 24 or 48 h, the cells in the upper chamber were scrubbed with cotton swabs, while the cells that migrated or invaded the lower chamber were fixed and colored using 4% paraformaldehyde and 0.1% crystal violet, respectively, and finally, pictures were captured using an inverted microscope.

### 2.7. AO/EB, AO, MDC Staining

HepG2 cells were planted on a 12-well plate overnight and stimulated with varying doses of ALR with or without 3-MA (2.5 mM) for another 24 h. After that, the cells were incubated separately with 5 *μ*L acridine orange (AO) solution (Sangon Biotech, Shanghai, China) or 5 *μ*L AO/EB (Sangon Biotech, Shanghai, China) at room temperature, protected from light for 5 min, or with 25 *μ*L monodansylcadaverine (MDC, Solarbio, Beijing, China) at room temperature, protected from light for 15 min. Finally, the images were photographed under an inverted fluorescence microscope.

### 2.8. mGFP-LC3-Adenovirus Staining

HepG2 cells were planted on a 12-well plate overnight and transduced with 10 MOI mGFP-LC3-adenovirus (HanBio, Shanghai, China) for 48 h. After that, the cells were stimulated with varying doses of ALR with or without 3-MA (2.5 mM) for another 24 h. Finally, the images were captured under an inverted fluorescence microscope.

### 2.9. Flow Cytometry Assay

HepG2 cells were planted on a 6-well plate overnight and then stimulated with varying doses of ALR with or without 3-MA (2.5 mM) for another 24 h. After that, the cells were treated with trypsin without EDTA and divided into two parts. One part was stained using 5 *µ*L annexin V-FITC and 5 *µ*L propidium iodide (KeyGen, Nanjing, China) for analysis of apoptosis. The other part was fixed in chilled 70% ethanol overnight, followed by incubation with 100 *μ*L RNase A solution and 400 *μ*L propidium iodide (Solarbio, Beijing, China) for analysis of the cell cycle by flow cytometry (Beckman, SA, USA).

### 2.10. Western Blot Assay

After treatment with varying doses of ALR with or without 3-MA (2.5 mM) for 24 h, HepG2 cells were collected and lysed, and the total protein concentration was detected by the BCA protein detection kit (KeyGen, Nanjing, China). Equal concentrations of protein were subjected to 12% SDS-PAGE gels and transferred to a PVDF membrane (Millipore, MA, USA). Then, the membrane was blocked with skim milk for 60 min and incubated with primary antibodies overnight at 4°C, followed by rinsing the membrane and incubating with secondary antibodies for 60 min. Finally, the protein bands were immersed in the enhanced chemiluminescence (ECL) substrate solution (Thermo, Carlsbad, USA) in the dark for 1 min and then analyzed using a protein chemiluminescence detector (Bio-Rad, USA). As a loading control, *β*-actin was detected simultaneously.

### 2.11. Statistical Analysis

All data in this study were analyzed with one-way analysis of variance (ANOVA) by using SPSS 26.0 (IBM, NY, USA), and were expressed as mean ± SD of three independent experiments. Differences were considered significant at *P*  <  0.05, *P*  <  0.01, and *P*  <  0.001.

## 3. Results

### 3.1. ALR Suppresses the Viability and Proliferation of HepG2 Cells

**Figure 1:**
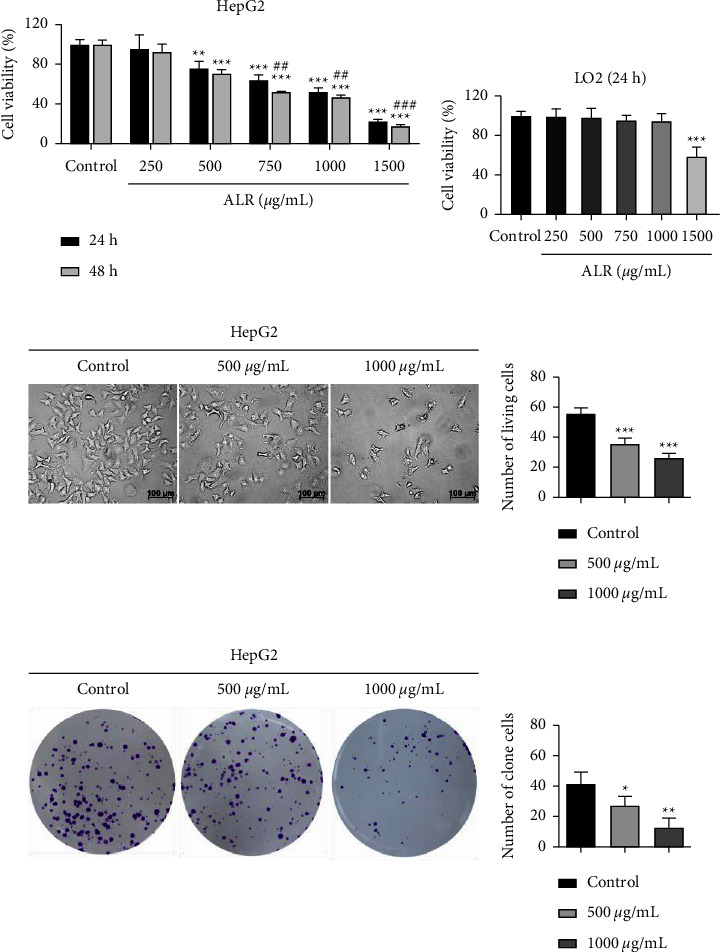
ALR suppresses the viability and proliferation of HepG2 cells. (a, b) CCK-8 assay to determine the cell viability. (c) Inverted microscopy to observe the cell morphological changes. (d) Colony formation assay to check the cell proliferation ability. ^*∗*^*P* <  0.05, ^*∗∗*^*P* <  0.01, and ^*∗∗∗*^*P* <  0.001, vs. the control group. ^##^*P* <  0.01 and ^###^*P* <  0.001, 48 h group vs. the 24 h group at the same concentration.

To investigate whether ALR affects the viability and proliferation of HepG2 cells, the CCK-8 and colony formation experiments were carried out. After treatment with ALR (0, 250, 500, 750, 1000 and 1500 *μ*g/mL) for 24 h and 48 h, CCK-8 results showed that the viability of HepG2 cells was suppressed in a time- and dose-dependent pattern (Figure 1(a)). Following 24 h of treatment, ALR at 500 *μ*g/mL or above had a significant inhibitory effect on HepG2 cell activity, while ALR at 1000 *μ*g/mL or below had no significant inhibitory effect on LO2 cells (Figures 1(a) and 1(b)). Therefore, the low concentration (500 *μ*g/mL) and high concentration (1000 *μ*g/mL) of ALR for 24 h were selected for follow-up experiments. The cell morphological changes were also observed by inverted microscopy, as shown in Figure 1(c). ALR treatment significantly decreased the cell numbers and adsorption capacity of HepG2 cells after 24 h, and the changes became more apparent with the increase in ALR concentration. In addition, the proliferation capacity of HepG2 cells was significantly inhibited in a dose-dependent pattern, as confirmed by the clone formation assay (Figure 1(d)). The above results suggest that ALR can suppress the viability and proliferation of HepG2 cells.

### 3.2. ALR Suppresses the Migration and Invasion of HepG2 Cells

**Figure 2:**
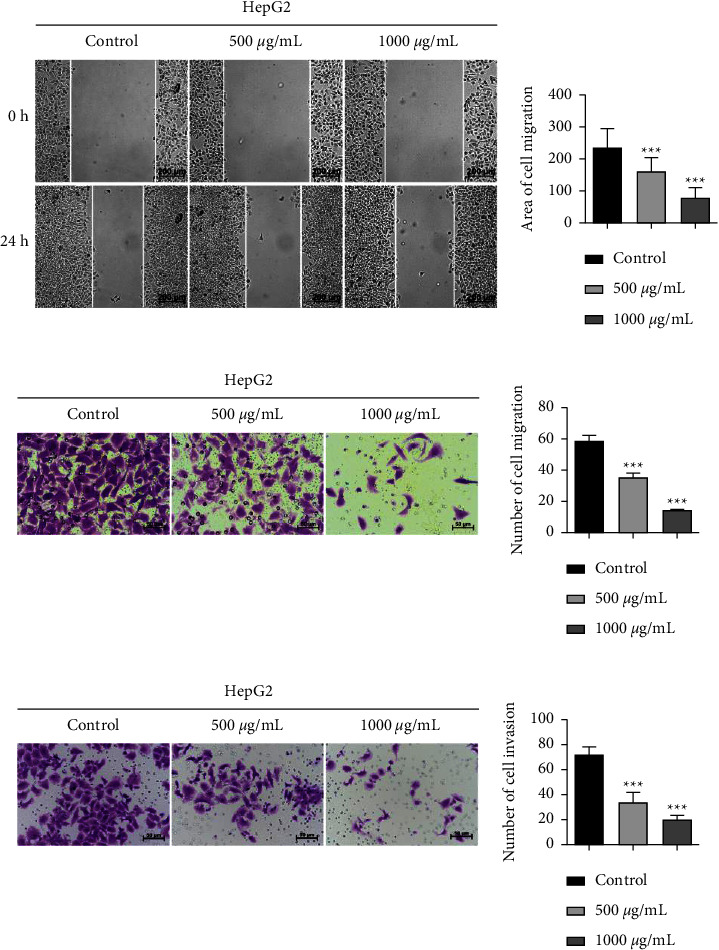
ALR inhibits the migration and invasion of HepG2 cells. (a) Cell scratch assay to evaluate the lateral migration ability. (b, c) Transwell assays without or with Matrigel to evaluate the vertical migration and invasion ability. ^*∗∗∗*^*P* <  0.001, vs. the control group.

To further evaluate the impact of ALR on the metastatic potency of HepG2 cells, the scratch assay, longitudinal migration, and invasion assays were performed. The scratch assay result (Figure 2(a)) showed that after 24 h of ALR treatment, the migration area on HepG2 cells was significantly decreased, and the migration ability was suppressed in a dose-dependent pattern. The transwell assays further indicated that the numbers of migratory and invasive cells were significantly reduced after ALR treatment for 24 h (Figures 2(b) and 2(c)). These findings reveal that ALR can inhibit the migration and invasion abilities of HepG2 cells.

### 3.3. ALR Promotes the Apoptosis and Cycle Arrest of HepG2 Cells

**Figure 3:**
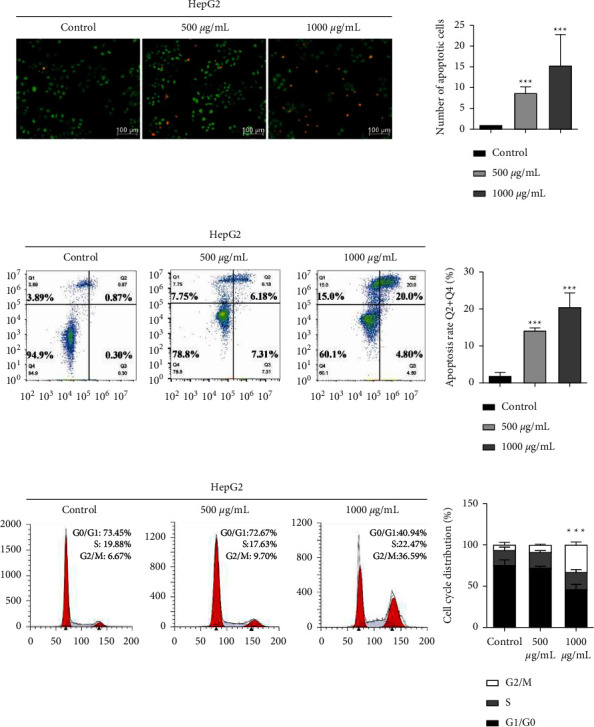
ALR promotes the apoptosis and cycle arrest of HepG2 cells. (a) AO/EB staining to detect the apoptosis cells. (b) V-FITC/PI staining to detect the apoptosis rate. (c) Flow cytometry assay to explore the cell cycle. ^*∗∗∗*^*P* <  0.001, vs. the control group.

To illustrate whether ALR inhibition of HepG2 cell proliferation is related to apoptosis and the cell cycle, we first detected the apoptosis rate by AO/EB and V-FITC/PI staining. The AO/EB result showed that abundant normal cells stained green in the control group, with intact nuclei and clear boundaries. However, with the increase of ALR concentration, some cells showed apoptosis characteristics such as chromosome shrinkage and fragmentation, and the numbers gradually increased, presenting bright orange (Figure 3(a)). The flow cytometry result further showed that the apoptotic rate of HepG2 cells increased significantly with the increase of ALR concentration, as shown in Figure 3(b). Next, the cell cycle was further measured, and in comparison with the control group, ALR at 500 and 1000 *μ*g/mL blocked the G2/M phase of HepG2 cells in a dose-dependent pattern (Figure 3(c)). The above results suggest that ALR can promote HepG2 cell apoptosis and G2/M phase cycle arrest in a dose-dependent pattern.

### 3.4. ALR Induces the Autophagy Morphological Changes of HepG2 Cells

**Figure 4:**
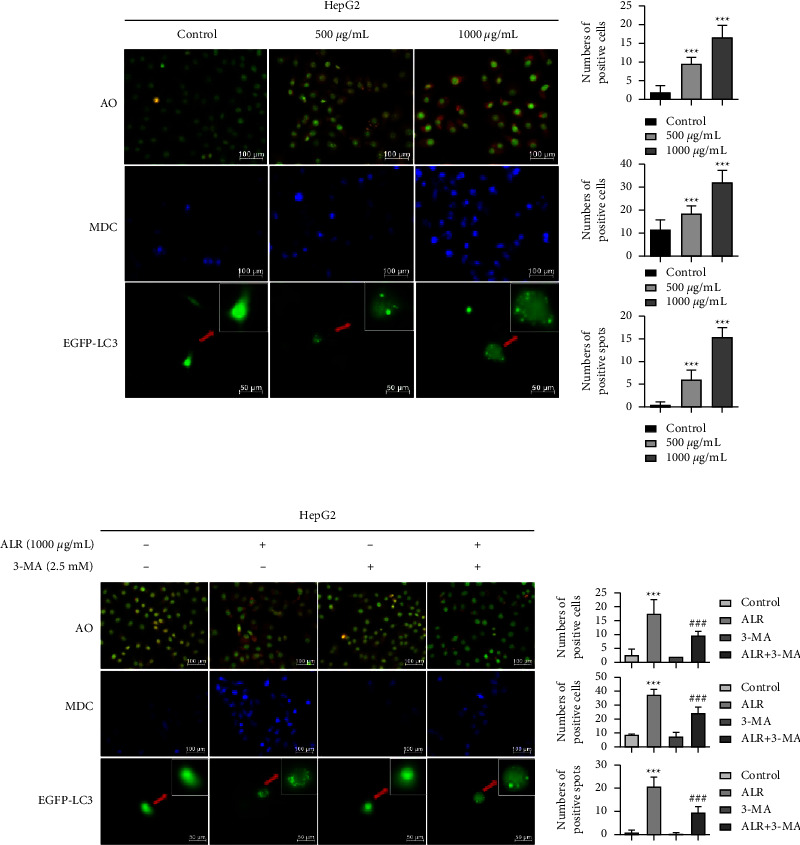
ALR induces the autophagy morphological changes of HepG2 cells. AO, MDC, and EGFP-LC3-adenovirus staining assays to determine the occurrence of autophagy without 3-MA intervention (a) and with 3-MA intervention (b) ^*∗∗∗*^*P* <  0.001, vs. the control group. ^###^*P* <  0.001, ALR vs. the ALR+3-MA group.

During tumorigenesis and development, autophagy plays a crucial role. To affirm whether ALR induces autophagy in HepG2 cells, the morphological changes of autophagy were detected by the following experiments: AO and MDC are two kinds of autophagic fluorescent dyes used to label autophagic vacuoles. When autophagy occurs, AO staining cells release orange-red fluorescence, while MDC staining cells show blue fluorescence under a fluorescence microscope. GFP-LC3-adenovirus is used to label and track LC3, which is an autophagosome indicator, and the number of green spots represents the strength of autophagic flow.  Figure 4(a) suggests that increasing the concentration of ALR significantly increased the number of fluorescent cells, indicating that ALR can induce the occurrence of autophagic flux.

To further confirm the autophagy of HepG2 cells induced by ALR, 3-methyladenosine (3-MA), which inhibits autophagy by blocking the formation of autophagosomes, was applied to observe the changes in autophagy's morphology. As shown in Figure 4(b), ALR combined with 3-MA treatment attenuated the number of fluorescent cells compared with the ALR group, as confirmed by the assays of AO staining, MDC staining, and GFP-LC3-Adenovirus detection. The above results suggest that ALR can induce autophagy morphological changes in HepG2 cells.

### 3.5. ALR Affects Autophagy-Related Proteins of HepG2 Cells

**Figure 5:**
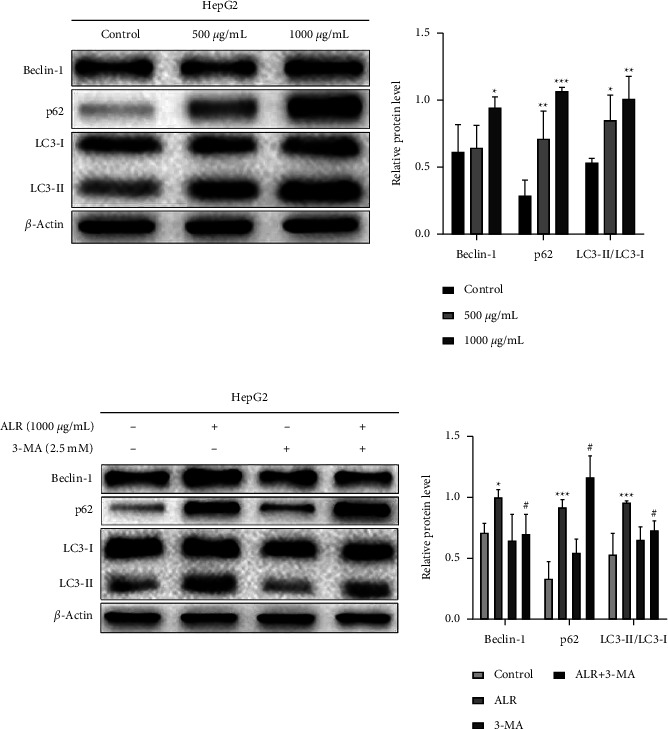
ALR affects the changes of autophagy-related proteins of HepG2 cells. Western blot assay to examine the protein changes of Beclin-1, p62, LC3-I, and LC3-II without 3-MA intervention (a) and with 3-MA intervention (b) ^*∗*^*P* <  0.05, ^*∗∗*^*P* <  0.01, and ^*∗∗∗*^*P* <  0.001, vs. the control group. ^#^*P* <  0.05, ALR vs. the ALR+3-MA group.

The occurrence of autophagy depends on the expression of related proteins; therefore, the protein levels of Beclin-1, LC3-I, LC3-II, and p62 were further examined by western blot before and after 3-MA intervention.  Figure 5(a) indicates that the Beclin-1, p62, and LC3-II/LC3-I were significantly upregulated with increasing ALR concentration. After intervention with 3-MA in HepG2 cells, compared with the ALR alone, ALR combined with 3-MA significantly downregulated Beclin-1 and LC3-II/LC3-I levels, whereas p62 was still upregulated (Figure 5(b)). The increase of p62 predicts that the cells may develop defective autophagy for the blockage, which will be further verified in later experiments. Taken together, the above results once again prove that ALR does cause autophagy in HepG2 cells.

### 3.6. Blocking Autophagy Impairs the Proliferative Activity and Metastasis Potential of ALR-Treated HepG2 Cells

**Figure 6:**
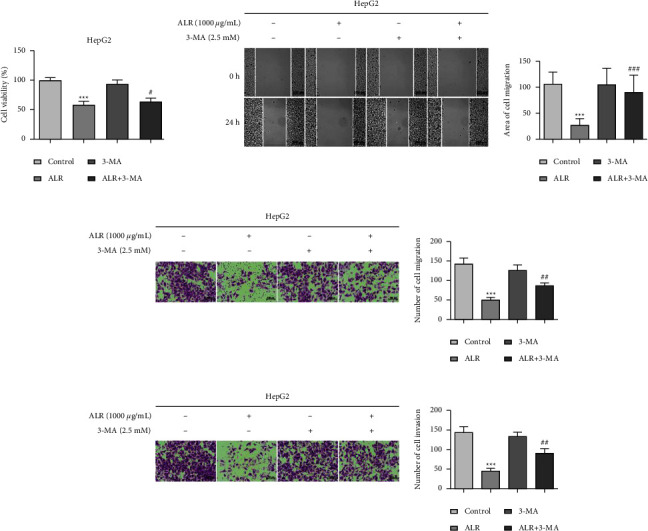
Blocking autophagy impairs the proliferative activity and metastasis potential of ALR-treated HepG2 cells. (a) CCK-8 assay to determine the cell viability. (b) Cell scratch assay to assess the lateral migration ability. (c, d) Transwell assays with or without Matrigel to evaluate the vertical migration and invasion capacity. ^*∗∗∗*^*P* <  0.001, vs. the control group. ^#^*P* <  0.05, ^##^*P* <  0.01, and ^###^*P* <  0.001, ALR vs. the ALR+3-MA group.

To reveal whether the inhibition of HepG2 cells activity and function by ALR is related to the occurrence of autophagy, the CCK-8, scratch assay, migration, and invasion assays were again carried out after HepG2 cells were intervened with 3-MA. Figures 6(a)–6(d) suggest that compared with the ALR alone, the proliferation viability, invasion, and migration abilities of the ALR combined with the 3-MA group were all reversed. These data suggest that autophagy exerts cytotoxic action, whereas inhibition of autophagy impairs ALR-induced viability, invasion, and migration of HepG2 cells.

### 3.7. Blocking Autophagy Impairs the Apoptosis and Cycle of ALR-Treated HepG2 Cells

**Figure 7:**
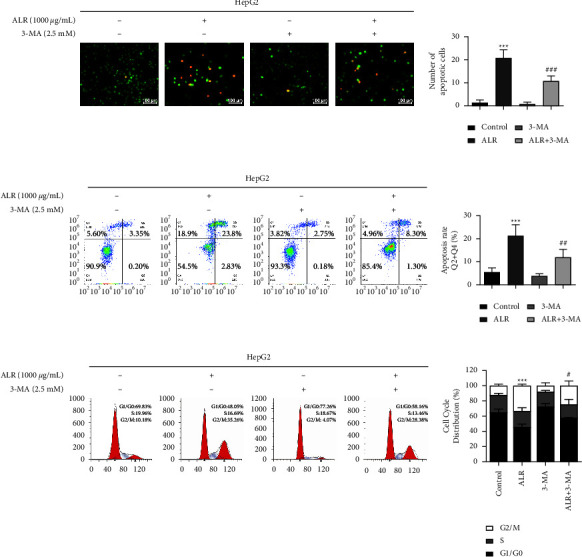
Blocking autophagy impairs the apoptosis and cycle of ALR-treated HepG2 cells. (a) AO/EB staining to detect the apoptosis cells. (b) V-FITC/PI staining to detect the apoptosis rate. (c) Flow cytometry assay to explore the cell cycle. ^*∗∗∗*^*P* <  0.001 vs. the control group. ^#^*P* <  0.05, ^##^*P* <  0.01 and ^###^*P* <  0.001, ALR vs. the ALR+3-MA group.

We have previously confirmed that the ALR can induce apoptosis, autophagy, and cycle arrest in HepG2 cells, but whether the apoptosis and cycle are related to autophagy is unknown. To this end, we explored the effects of inhibiting autophagy on apoptosis and the cell cycle. As shown in Figures 7(a) and 7(b), the apoptotic cells in the ALR combined with 3-MA treatment group were less significant than those in the ALR alone group, as confirmed by the assays of AO/EB staining and V-FITC/PI staining. A similar trend was observed that the percentage of G2/M phase in the combined group was reversed compared with the ALR group (Figure 7(c)). The above results show that the ALR regulates apoptosis and cycle of HepG2 cells through autophagy.

### 3.8. ALR Regulates Autophagy of HepG2 Cells by AMPK/mTOR Pathway

**Figure 8:**
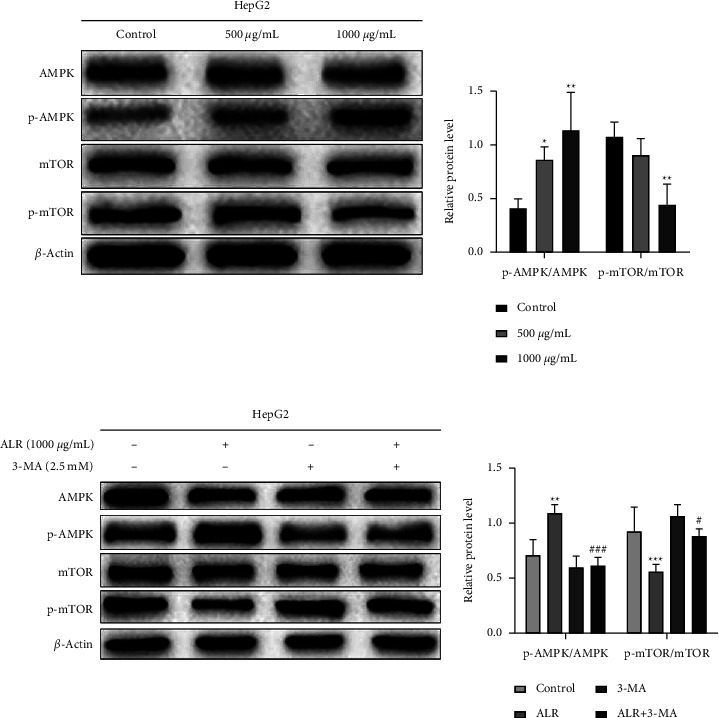
ALR regulates autophagy of HepG2 cells by the AMPK/mTOR pathway. Western blot assay to analyze the protein changes in p-AMPK, AMPK, p-mTOR, and mTOR without 3-MA intervention (a) and with 3-MA intervention (b) ^*∗*^*P* <  0.05, ^*∗∗*^*P* <  0.01, and ^*∗∗∗*^*P* <  0.001, vs. the control group. ^#^*P* <  0.05 and ^###^*P* <  0.001, ALR vs. the ALR+3-MA group.

Considering the important role of the AMPK/mTOR signaling pathway in autophagy, we examined by western blot whether this cascade is involved in ALR-induced autophagy. As illustrated in Figure 8(a), in comparison with the control group, with the increase in ALR concentration, the p-AMPK/AMPK protein level was markedly upregulated, while the p-mTOR/mTOR level was significantly downregulated. However, the p-AMPK/AMPK and p-mTOR/mTOR protein levels were all reversed in the ALR combined with 3-MA group compared with the ALR alone group (Figure 8(b)). Taken together, these results reveal that ALR can induce autophagy in HepG2 cells by activating the AMPK/mTOR signaling pathway.

## 4. Discussion

In recent years, with the enhancement of health consciousness, people have paid more and more attention to dietary intervention for cancer prevention and management, and some natural products with wide sources and low or no toxicity have shown promising effects in this regard [18, 19]. LR (also called black goji berry) is a wild prickly shrub, which is mainly planted in northwestern China such as Ningxia, Qinghai, and Xinjiang due to its unique drought-resistant and salt-resistant characteristics [20]. The fruit of LR, with a violet-black appearance, has been broadly utilized in China as nutraceutical food and folk medicine for at least 2000 years [21]. LR contains a variety of bioactive ingredients, e.g., flavonoids, anthocyanins, polysaccharides, alkaloids, phenolic acids, etc., which make it exhibit anti-inflammatory, antitumor, antiaging, anti-infertility, and other biological functions [7]. LR contains a large amount of anthocyanins, a class of water-soluble flavonoid compound widely existing in plants, and is an ideal plant source for anthocyanin extraction [6]. The basic structural unit of anthocyanins is flavylium, and the main components include pelargonidin, cyanidin, delphinidin, petunidin, malvidin, etc. [22]. Although multiple studies have demonstrated the anticancer effects of anthocyanins extracted from strawberries, grapes, corn, etc. [8], there is still a lack of investigation on the anti-HCC activity and exact molecular mechanism of ALR. Therefore, in this research, we first investigated the effects of ALR on the proliferation, migration, and invasion of HepG2 cells. Our results (Figures 1 and 2) verified that ALR significantly suppressed the viability, clone formation, migration, and invasion of HepG2 cells in a dose-dependent pattern, which showed consistent biological functions with other sources of anthocyanins [11]. A study reported that apoptosis induction and cycle arrest were closely related to tumor proliferation inhibition [23]. Therefore, we further analyzed the apoptosis and cycle of ALR-treated HepG2 cells. The result suggested that the apoptotic cells significantly increased with increasing ALR concentration, and the cells were arrested in the G2/M phase, as indicated by AO/EB staining, V-FITC/PI staining, and cycle analysis (Figure 3). Li et al. also demonstrated that anthocyanins derived from *Lycium ruthenicum* Murray induced apoptosis in DU-145 prostate cancer cells [24]. However, studies found that anthocyanins have different targets on the cell cycle for different cancer cells. For example, anthocyanins from blue corn and tortillas could arrest the cycle of breast and prostate cancer cells at the G1 phase [11].

Research has demonstrated that autophagy plays a pivotal role in regulating cell survival [25]. Autophagy is a self-protective mechanism for cells to adapt to various environmental changes and maintain homeostasis [26]. The occurrence of autophagy is regulated by a series of autophagy-related proteins [27]. For example, Beclin-1 participates in the beginning of autophagy and the maturation of autophagosome [28]. During the autophagosome formation, LC3-I is transformed to LC3-II, and the LC3-II/LC3-I ratio is usually used to assess autophagy levels [29]. The autophagy substrate p62 is fused into the autophagosome by interacting with LC3 and then gradually degraded by the autophagolysosome [30]. A variety of natural compounds, such as silibinin and resveratrol, have been proven to promote autophagy in various cancer cells [31, 32]. We also discovered a significant aggregation of autophagic vacuoles and autophagosomes with the increase in ALR concentration, as confirmed in Figure 4. Furthermore, the Beclin-1, p62, and LC3-II/LC3-I protein levels were significantly increased in ALR-treated HepG2 cells (Figure 5). Notably, p62, an autophagy negative regulator, was not reduced; instead, p62 expression was significantly upregulated in ALR-treated HepG2 cells, especially when ALR was combined with 3-MA (Figure 5(b)). This phenomenon indicated that ALR blocked autophagy flux, which meant autophagosomes were formed but had not been degraded. Clinical studies have confirmed that aggregation of p62 could lead to poor prognosis for HCC patients [33]. Studies suggested that the aggregation of p62 could activate the Nrf2 antioxidant system and resist ferroptosis in liver cancer cells, thereby promoting the growth of HCC [34]. Whether ALR-induced p62 aggregation is related to ferroptosis remains to be further explored.

Given that the role of autophagy in tumors sometimes involves paradoxical functions, we further analyzed the relationship between autophagy and antitumor activity. We observed that combined treatment with 3-MA attenuated the proliferation inhibition, invasion, migration, apoptosis, and G2/M phase cycle block of ALR-induced HepG2 cells, as shown in Figures 6 and 7, suggesting that autophagy plays a cytotoxic role. However, Chen et al. found that autophagy inhibition promoted the apoptosis and antiproliferative activity of delphinidin-induced breast cancer HER-2 cells, suggesting that delphinidin induces protective autophagy [17]. These studies indicate that anthocyanin-induced autophagy plays different roles in different tumors. To further illuminate the mechanism of autophagy regulation, in view of the crucial role of mTOR in regulating autophagy, we examined the expression levels of mTOR and its upstream molecule AMPK. Our results confirmed that ALR induces autophagy in HepG2 cells by activating AMPK and inhibiting the mTOR pathway (Figure 8). A similar finding was reported that meoru anthocyanins, activators of AMPK *α*1, could prevent colon cancer cell growth by inhibiting mTOR phosphorylation [35]. To summarize, this work sheds light on the anticancer effects of ALR and suggests that it may have therapeutic potential as an autophagy inducer in HepG2 cells. However, the susceptibility to oxidation and low bioavailability of anthocyanins restrict their anticancer potential and clinical application. Nonetheless, ALR is still highly recommended as a nutritional supplement for cancer prevention and management without side effects.

## 5. Conclusions

**Figure 9:**
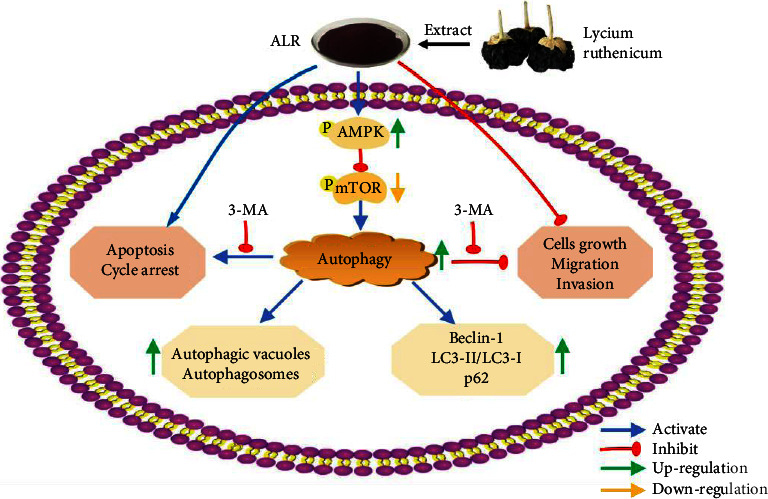
Schematic model of ALR-induced antitumor activity and molecular mechanisms of HepG2 cells. ALR inhibits HepG2 cell growth, migration, and invasion and promotes apoptosis and G2/M cycle arrest by activating the AMPK/mTOR autophagy signaling pathway.

Taken together, our study, summarised in Figure 9, clearly confirms that ALR induces autophagy via the AMPK/mTOR pathway and that autophagy inhibition impairs the proliferation inhibition, invasion, and migration capabilities as well as apoptosis and G2/M phase arrest of ALR-treated HepG2 cells. The in vivo anti-HCC effect of ALR will require further evaluation and exploration. In a word, ALR may be considered a promising natural product for the design of novel anticancer agents.

## Data Availability

The data used to support the findings of this study are available from the corresponding authors upon request.
